# Influence of Dielectric Barrier Discharge Cold Plasma Treatment on Starch, Gelatin, and Bacterial Cellulose Biodegradable Polymeric Films

**DOI:** 10.3390/polym14235215

**Published:** 2022-11-30

**Authors:** Mayara Lima Goiana, Adriano Lincoln Albuquerque Mattos, Henriette Monteiro Cordeiro de Azeredo, Morsyleide de Freitas Rosa, Fabiano André Narciso Fernandes

**Affiliations:** 1 Departamento de Engenharia Química, Universidade Federal do Ceará, Fortaleza 60440-900, CE, Brazil; 2 Embrapa Agroindustria Tropical, R. Dra. Sara Mesquita, 2270, Fortaleza 60511-110, CE, Brazil; 3 Embrapa Instrumentação, R. 15 de Novembro, 1452, São Carlos 13560-970, SP, Brazil

**Keywords:** biopolymer, edible films, starch, gelatin, bacterial cellulose

## Abstract

The environmental damage caused by plastic packaging and the need to reduce pollution requires actions to substitute plastic materials for more sustainable and biodegradable materials. Starch, gelatin, and bacterial cellulose films are three potential biodegradable polymeric films for use in packaging. However, these materials need improvements in their physical, chemical, and mechanical properties to be used in packaging. In this work, these films were treated with cold plasma to evaluate the effects of treatment conditions on several physical, chemical, and mechanical properties. The dielectric barrier discharge plasma technology was applied with varying treatment times (0 to 20 min) and excitation frequencies (50 to 900 Hz) at 20 kV. The optimal excitation frequency for starch films (50 Hz) was different from the optimal frequency for gelatin and bacterial cellulose films (900 Hz), indicating a high dependency on the treatment in this variable that is often neglected. Plasma treatment improved the hydrophobicity, surface morphology, water resistance, and mechanical properties of all three films, with the advantage of not recurring to chemical or biological additives.

## 1. Introduction

Environmental damage caused by the accumulation of non-biodegradable plastic packaging waste has generated high interest in developing possible biodegradable substitutes. Natural biodegradable polymers-based packaging is especially demanded by the food industry, which is one of the largest consumers of plastic packaging [1].

Biomolecules such as starch, bacterial cellulose, and gelatin have been used to develop packaging materials [2]. These packaging materials are considered biodegradable, sustainable, and environmentally friendly [3,4]. However, some disadvantages must be overcome, such as their high hydrophilicity, low water vapor, gas, and light barrier, and low mechanical resistance [1,5].

Starch can be obtained from several vegetable sources, such as maize, seed kernels, cereal grains, roots, and fruits. Starches are biodegradable and usually have low costs. It consists of two fractions: amylose (straight chain) and amylopectin (branched chain). Starches have good film-forming properties but present some disadvantages, including low mechanical performance and high hydrophilicity, which result in rapid moisture absorption during use and storage [6].

Bacterial cellulose is a natural nanostructured polymer produced by several bacteria. It has good thermal stability, high porosity, good mechanical properties, amphiphilic character, and a high liquid sorption capacity [7]. However, it has a high permeability to water vapor, which limits its application [8].

Gelatin is a linear protein resulting from collagen denaturation of bovine, swine, or fish origin. It has a good ability to form films; however, it has a limited moisture barrier property [9].

Improvements on polymeric films can be carried out by several techniques, such as cold plasma [10], ultrasonics [11], doping with additives [12], production of composites [13], and other procedures. In this study, we have applied cold plasma technology to induce changes to starch, gelatin, and bacterial cellulose films.

Cold plasma is a technology that can be applied for surface treatment, including modification or functionalization of polymers. Recently, it has emerged as an innovative, effective, and sustainable alternative to traditional chemical treatments. One of the plasma generation methods is the dielectric barrier discharge (DBD), which operates at room temperature and atmospheric pressure and does not involve using petroleum-based solvents. Cold plasma generates ultraviolet light, high-energy electrons, and reactive particles, which can initiate several physical-chemical reactions on the surface of polymers [14,15].

Several studies have investigated the effect of DBD plasma on polymers, varying the exposure time, voltage, and excitation frequency. Wu et al. [15] showed that cold plasma improved casein-based films’ barrier and mechanical properties by increasing the plasma generation voltage. Films from corn starch with high amylose content and treated with high-voltage cold plasma (70 and 80 kV) increased the film roughness and hydrophilicity [16]. New functional groups containing oxygen were introduced to the sodium caseinate film surface, improving its hydrophilicity by applying short plasma exposure times (1 to 5 min, at 70 kV). At the same time, no changes occurred in the helical structure of the protein [17].

Studies on plasma treatment of bacterial cellulose films are still scarce and more work is needed to understand the changes induced by plasma species on the properties of bacterial cellulose films. Studies have been carried out on the degradation of toxic compounds from the raw material used as a carbon source for bacterial cellulose production [18,19] and for the deposition of oxides and other materials on the surface of bacterial cellulose [20]. However, there are no studies on plasma treatment of bacterial cellulose films aiming toward the improvement of its physical, chemical, and mechanical properties.

Modification of starch using plasma technology has been successfully addressed. Plasma increases carbonyl and carboxyl groups, resulting in improved paste viscosity, hydration properties, and digestibility [21,22]. These studies showed that the changes in starch properties were greater at excitation frequencies between 100 and 300 Hz and not at 50 Hz as commonly applied [10,21]. Plasma treatment in starch films also indicated that the optimal excitation frequency range was between 200 and 300 Hz [10,23].

Most studies on cold plasma treatment on polymers were carried out at a single plasma excitation frequency, usually at 50 Hz. However, the excitation frequency has been proven to be an essential factor in cold plasma applications [24,25,26]. The excitation frequency influences the concentration and type of ions and free radicals generated in the cold plasma, inducing different chemical transformations in the treated product. Plasma treatments on bacterial cellulose and gelatin films at 50 Hz have not resulted in good improvements in film properties. Due to the importance of the excitation frequency in the generation of plasma in many applications, such as starch modification [10,21], aroma modulation [24], phenolics content [27], and mitigation of off-flavors [28], this work has addressed the significance of the excitation frequency in three different biodegradable polymer films.

This study applied DBD plasma at three different plasma excitation frequencies to three films produced from natural polymers (gelatin, starch, and bacterial cellulose). The effects of cold plasma treatment on the film water solubility, hydrophobicity, chemical group composition, mechanical properties, and surface morphology were evaluated.

## 2. Materials and Methods

### 2.1. Materials

Commercial corn starch (Unilever, brand Maizena™, Garanhuns, Brazil) and commercial fish gelatin (Fitoway Laboratório Nutricional Ltd. a, brand São Pedro™, Tarumã, Brazil) were purchased in the local supermarket. Bacterial cellulose membranes were supplied by Seven Indústria de Produtos Biotecnológicas Ltd. (Ibiporã, Brazil). Glycerol was purchased from Dinâmica (São Paulo, Brazil).

### 2.2. Elaboration of the Biopolymer Films

The casting technique was used in the production of the films. Glycerol acted as a plasticizer. The degassing step of the filmogenic dispersions to remove the air bubbles formed during the process, common to the three films, was carried out before the final drying. The process took place in a vacuum system with a Vacuubrand-1C pump.

The starch film was produced following the methodology proposed by Oliveira et al. [29]. In brief, 5 g of corn starch was dissolved in 100 mL of distilled water (5% *w*/*v*) and heated to 95 °C under magnetic stirring for 30 min for complete starch gelatinization. Glycerol was added to the starch solution in a 25% *w*/*w* proportion and maintained at 60 °C for 15 min under magnetic stirring. Afterward, the dispersion was homogenized in an Ultra-Turrax (IKA model T25) at 10,000 rpm for 15 min. The degassed filmogenic dispersion was poured onto glass plates coated with a polyester film (Mylar™) and allowed to dry under ambient conditions (25 °C) for 24 h.

Bacterial cellulose membranes were oven-dried for 48 h at 50 °C and ground in an analytical mill (IKA model A11). The dried bacterial cellulose underwent an oxidation process following the method presented by Saito et al. [30]. After oxidation, a mechanical treatment took place in a colloid mill (Meteor model Rex Inox I-V-N) for 10 min, obtaining nano-fibrillated bacterial cellulose (NFBC). The NFBC was freeze-dried (Liotop model LP510). The methodology proposed by Nascimento et al. [31] was used to obtain the filmogenic dispersion of bacterial cellulose. For this purpose, an aqueous dispersion was prepared to contain 1% (*w*/*v*) of lyophilized NFBC and 50% (*w*/*w*) of glycerol on a dry basis of the matrix. The dispersion was homogenized in an Ultra-Turrax (IKA model T25) at 13,000 rpm for 15 min, sonicated for 2 min at 19 kHz (Unique model DES500) for degassing, and dried in stainless-steel trays coated with Mylar™ in an oven at 50 °C for 48 h.

Gelatin films were prepared following the procedure described by Santos et al. [32]. An aqueous solution was prepared with 9.6% *w/v* gelatin and 25% *w/v* glycerol (on a dry basis). The gelatin was hydrated and heated to 50 °C for 15 min, adding the glycerol in the last 5 min, under constant agitation. The solution was homogenized in an Ultra-Turrax (IKA model T25) at 10,000 rpm for 10 min. The degassed film-forming solution was poured onto glass plates covered with Mylar™ and allowed to dry at 25 °C for 24 h.

### 2.3. Dielectric Barrier Discharge Plasma Treatment

The films were treated with cold plasma in dielectric barrier discharge equipment consisting of a power source (Inergiae model PLS0130) and two 8 cm-diameter aluminum electrodes separated by 2 mm acrylic plates. The films were placed within a 3 cm gap between the dielectric barriers centered with the electrodes.

Cold plasma was generated at 20 kV (maximal voltage achieved with the equipment) and at excitation frequencies of 50, 400, and 900 Hz. The films were treated for 5 min. The films were treated adhered to the polyester support (Mylar^TM^) and without the polyester support. All assays were carried out in triplicates.

The optimal excitation frequency for plasma treatment was defined as the frequency at which greater changes were observed in the chemical composition, hydrophobicity, and film solubility. The best films regarding hydrophilicity and solubility were plasma-treated for 10, 20, and 30 min under the optimal excitation frequency for further analysis of the films’ water solubility, hydrophobicity, and chemical group composition. The films with the best results for hydrophobicity, water solubility, and chemical composition were further tested for changes in surface morphology and mechanical properties.

### 2.4. Physical and Chemical Analysis

#### 2.4.1. Thickness and Humidity

The thickness of the films was measured using a digital micrometer (Mitutoyo model IP65). Ten replicates were taken.

The film humidity was measured in an infrared moisture balance (Marte model ID50) operating at 100 °C. The humidity was based on 1 g of sample. Three replicates were taken.

#### 2.4.2. Hydrophobicity

Changes in the hydrophobicity of the films were determined through the water contact angle (GBX Instrumentation Specifique), attained according to ASTM D-5725-99 [33]. Film samples (2 × 2 cm) were fixed to a glass holder, and an image was captured (Pixe Link Nikon camera) when the drop touched the film surface. The test was carried out at room temperature (23 ± 2 °C). All assays were performed in quadruplicate.

#### 2.4.3. Water Solubility

The water solubility was determined following the method described by Pena-Serna and Lopes-Filho [34]. The film samples (2 cm-diameter discs) were dried in an oven at 105 °C for 24 h, weighed (*W*_0_), and immersed in 50 mL of distilled water at 25 ± 2 °C for 24 h under agitation in an orbital shaker (Tecnal model TE-142) at 100 rpm. After this period, the samples were removed and dried in an oven (105 °C for 24 h) to determine the mass of the material that was not solubilized (*W*). The solubility was calculated using Equation (1):

(1) $$S\left(\%\right)=\frac{W-W_{0}}{W_{0}}100$$

#### 2.4.4. FTIR Analysis

The absence or formation of chemical groups on the surfaces of the films was evaluated using Fourier transform infrared (FTIR) spectra. A Perkin-Elmer Spectrum Two equipment (Perkin-Elmer, Waltham, MA, USA) was used in attenuated total reflection mode (ATR). The spectra were collected in wavelengths between 4000 and 650 cm^−1^, using 32 scans and a 4 cm^−1^ resolution.

### 2.5. Surface Morphology

The surface morphology was characterized using a Quanta FEG SEM at an accelerating voltage of 10 kV. The films were cut into 5 × 5 mm squares, fixed on the sample table with conductive tape, and covered with a thin layer of gold.

### 2.6. Mechanical Properties

The mechanical properties of the films were determined using an EMIC DL-3000 equipment, following the standard method D882-18 [35]. The tensile strength (TS), elongation at break (EB), and elasticity module (EM) were determined. The films were cut into 4 × 75 mm dumbbell shapes. The mechanical tests were performed with an initial clamping distance of 50 mm and a tensile rate of 50 mm/s. All measurements were carried out in quintuplicate.

### 2.7. Selection of the Best Films and Cold Plasma Operating Conditions

The selection of the best biodegradable polymeric films and their respective cold plasma operating conditions was based on a desirability function that considered the highest change in functional groups measured by FTIR, highest hydrophobicity, and lowest water solubility (Equation (1)). These three factors were chosen because the goal of cold plasma processing is to induce physical and chemical changes in the film surface and chemical structure, and because films with high hydrophobicity and low water solubility are desired for food applications. The weight of the hydrophobicity in the desirability function was higher than the other two factors due to the importance of this property in food packaging. The contact angle divided by 90° was used to address the hydrophobicity, measuring how close the contact angle was from the value for which a film is considered hydrophobic. For the changes in the chemical structure, the absorbance that presented the largest change was used in the function.

(2) $$Score=\frac{\left(100-S\left(\%\right)\right)}{100}+\frac{\left(Abs^{mas}-Abs\right)}{Abs^{max}}+2\frac{CA}{90}$$

The starch, gelatin, and bacterial cellulose with the highest scores were considered the best biodegradable films. Surface analysis and mechanical properties’ analysis were carried out only with the best films.

### 2.8. Statistical Analysis

The results were presented as mean ± standard deviation. The statistical analysis was performed using analysis of variance (ANOVA), followed by the Tukey’s test, with the Statistica version 7 software.

## 3. Results

The films were produced by the casting method, which enabled attaining films with low thickness and humidity (Table 1). The starch and gelatin films were thinner than the bacterial cellulose film, while their humidity was not significantly different.

### 3.1. Hydrophobicity

Table 2 presents the effects of the excitation frequency on the hydrophobic character and water solubility of the films subjected to plasma treatment.

The hydrophobicity of the film is an essential feature in deciding the packaging application of any polymeric film [16]. A totally hydrophobic film is characterized by contact angles equal to or greater than 90° [14]. Among the films tested, the bacterial cellulose film had the highest hydrophobicity, while the starch film had the lowest hydrophobicity.

The Mylar support acted as an extra barrier between the electrodes and the films, reducing the effects of plasma treatment. The hydrophobicity of the films did not change significantly or decrease when the Mylar support was used. Thus, its use is not recommended when applying cold plasma.

Plasma treatment increased the hydrophobicity of the bacterial cellulose and gelatin films, while no significant changes were observed in starch films when the Mylar support was not used. The highest increase in hydrophobicity was observed at a plasma excitation frequency of 900 Hz. At 50 Hz, no significant change was observed. Thus, proper setting of the excitation frequency is critical to increasing the hydrophobicity of the films.

The films were subjected to plasma treatment for an extended period at the optimal excitation frequency of each film (Table 3). The increase in processing time increased the contact angle of all films, indicating the production of films with higher hydrophobicity. Thus, the processing time was a significant variable for the process (*p* < 0.05). Plasma application increased the hydrophobicity of the films by 15%, 6%, and 20%, respectively, for the starch, bacterial cellulose, and gelatin films.

The increase in hydrophobicity has been attributed to changes in the surface morphology of the films due to an increase in surface roughness. This will be discussed in Section 3.4.

Table 4 presents the contact angles of several films reported in the literature. Starch films have a low contact angle (between 20 and 56°) and are distant from the recommended 90° contact angle for hydrophobic surfaces. Plasma processing and high-pressure processing increase the hydrophobicity of starch films, but these physical changes have limited effects on the films’ hydrophobicity. Chemical additives such as cinnamaldehyde have a higher impact on the contact angle and the hydrophilic nature of starch films.

Plasma treatment increased the contact angle of gelatin films by 17%, while the production of a gelatin + chitosan composite reduced the contact angle [38].

The use of plasma treatment was shown to increase and decrease the contact angle. Proper setting of the excitation frequency (900 Hz) improved the contact angle, while low excitation frequencies (50 Hz) decreased the contact angle, worsening the wettability properties of gelatin films [39]. This comparison evidences the importance of the excitation frequency in plasma treatment of bacterial cellulose films.

### 3.2. Solubility in Water

The solubility of the films is an important property that can indicate the presence of hydrophilic groups, in addition to assessing the resistance in aqueous media [41]. Overall, the starch film is the most soluble film, and the bacterial cellulose film is the least soluble film (Table 2).

The Mylar support has also attenuated the effects of plasma treatment regarding the solubility of the films. The solubility of the bacterial cellulose and gelatin films increased when the support was used, which is not a desirable result.

On the other hand, the solubility of all films decreased when the Mylar support was not used. The highest decrease in solubility was observed for the bacterial cellulose film, which was already the least soluble film.

The excitation frequency affected each film differently. A lower excitation frequency (50 Hz) decreased the solubility of the starch film, while a higher excitation frequency (1000 Hz) decreased the bacterial cellulose and the gelatin film solubilities.

The films were subjected to plasma treatment for an extended period at the optimal excitation frequency of each film (Table 5). The increase in processing time decreased the solubility, improving its potential application in packaging. Processing time was also a significant variable for solubility (*p* < 0.05). Plasma application reduced the solubility of the films by 6%, 40%, and 18%, respectively, for the starch, bacterial cellulose, and gelatin films.

Although an 80% increase was observed in the insoluble matter of the starch film after plasma treatment, this film remained with an insoluble matter below that recommended for food packaging applications [42]. The potential application of a film in the food industry requires water-insoluble packaging to increase product integrity and water resistance [42].

Table 6 presents the water solubility of several films reported in the literature. Starch films have an extensive range of solubility (between 15% and 98%), which is usually related to the amylose–amylopectin ratio in the starch. Plasma processing decreased the water solubility of starch films more than adding chemical additives such as cinnamaldehyde and orange peel, while having a similar effect as the starch/date palm pits composite.

Plasma treatment decreased the solubility of bacterial cellulose by 40%, which was a similar result attained by chemical treatment to convert bacterial cellulose into nanocellulose (48%) and nanocrystalline cellulose (38%). However, the decrease achieved with plasma treatment reduced the bacterial cellulose to the lowest level reported in the literature. The physical and chemical treatments of bacterial cellulose reduced the solubility more than the formation of composites, for example, the whey protein + bacterial cellulose composite [46].

Gelatin films were less affected by plasma treatment than by enzymatic treatment and incorporation of chemical additives. Enzymatic treatment of gelatin films with transglutaminase [48] showed a slightly superior effect in decreasing the film solubility (25%) compared to the plasma treatment (18%). The addition of polyvinyl alcohol was reported as a good alternative to reduce the gelatin film solubility [47].

### 3.3. FTIR Analysis

**Figure 1:**
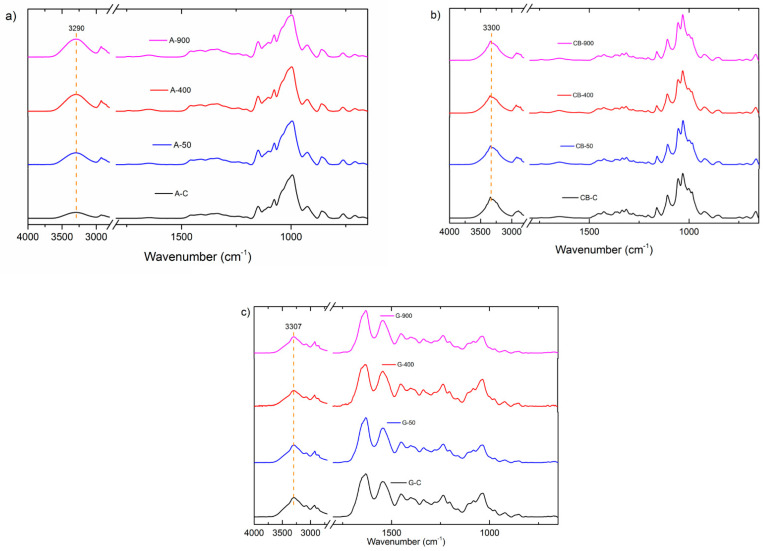
FTIR spectra of (**a**) starch films, (**b**) bacterial cellulose films, and (**c**) gelatin films subjected to plasma treatment.

The FTIR spectra of the plasma-treated films are shown in Figure 1. The spectra of the starch film presented the characteristic peaks at 3284 cm^−1^ (-OH vibration), 2900 cm^−1^ (-CH vibration), 1150 cm^−1^ (C-O stretching), 1076 cm^−1^ (C-H bending), and 929 cm^−1^ (C-H out-of-plane bending).

The spectra of the bacterial cellulose film presented the characteristic peaks at 3300 cm^−1^ (-OH vibration), 2900 cm^−1^ (-CH vibration), 1160 cm^−1^ (C-O-C asymmetric stretching vibration), and 1030 cm^−1^ (C-O stretching). The spectra of the gelatin film presented the characteristic peaks at 3300 cm^−1^ (-OH vibration), 2900 cm^−1^ (-CH vibration), 1635 cm^−1^ (C = O stretching vibration), 1550 cm^−1^ (N-H bending vibration), 1160 cm^−1^ (C-O-C asymmetric stretching and NH_2_ oscillation vibrations), and 1030 cm^−1^ (C-O stretching).

No new bands corresponding to the formation of chemical clusters were observed in the plasma-treated starch, bacterial cellulose, and gelatin films. Thus, plasma treatment promoted the interaction between the reactive plasma species and the functional groups already in the films [49].

The hydroxyl band (3284 cm^−1^) showed a slight reduction in intensity in all three films. The most significant reductions occurred at 50 Hz for the starch film and 900 Hz for the bacterial cellulose and gelatin films. All other characteristic bands of the films were preserved. The hydroxyl band further reduced its intensity when treated for an extended period (20 min). This reduction can be correlated to the higher hydrophobicity of the material plasma-treated for 15 and 20 min.

### 3.4. Best Biodegradable Films and Plasma Operating Conditions

The results obtained for hydrophobicity, water solubility, and FTIR analysis for the films processed at different cold plasma frequencies were evaluated using the desirability function to determine the best plasma conditions for each biodegradable film. Table 7 presents the scores obtained for the desirability function.

The scores of the desirability function have pointed out that the best starch film was produced at 50 Hz, while the best bacterial cellulose and gelatin films were produced at 900 Hz. These scores corroborate to point out that the cold plasma excitation frequency is an important variable when applying plasma to polymeric films.

The highest scores for each film agreed with the highest changes observed in hydrophobicity, water solubility, and chemical changes. Among the three films tested herein, the bacterial cellulose film presented the highest scores, especially due to its highest hydrophobicity, which is an important property of biodegradable films for food packaging.

### 3.5. Surface Morphology

**Figure 2:**
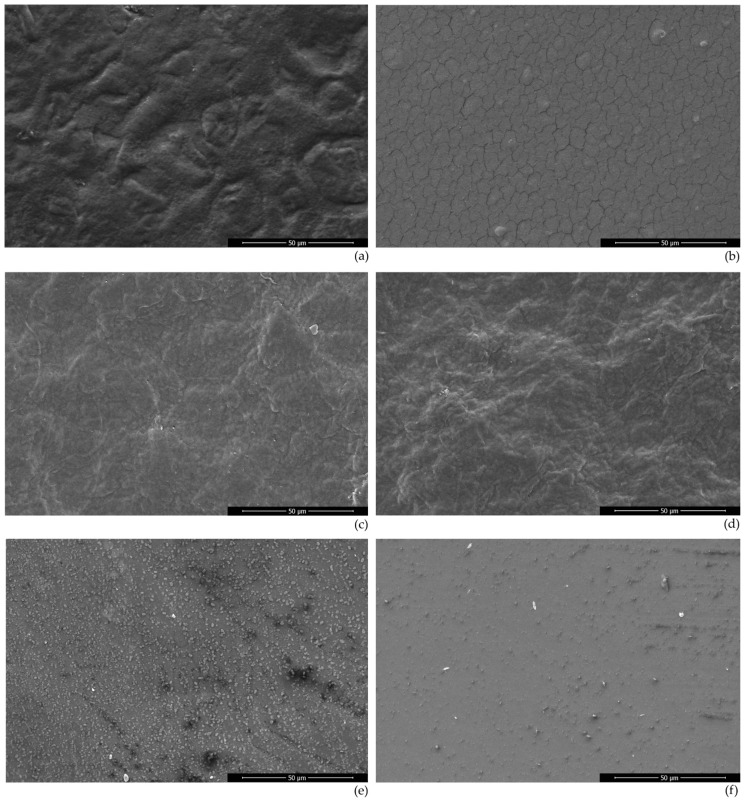
Scanning electronic micrographs of: (**a**) untreated starch film, (**b**) plasma-treated starch film (20 min at 50 Hz), (**c**) untreated bacterial cellulose film, (**d**) plasma-treated bacterial cellulose film (20 min at 900 Hz), (**e**) untreated gelatin film, and (**f**) plasma-treated gelatin film (20 min at 900 Hz).

The starch film showed a rough surface with no cracks (Figure 2). Plasma processing significantly changed the surface of the film. The surface became smoother than the untreated film, but many microscopic cracks appeared on the surface. Tiny white dots appeared scattered throughout the film surface, which could be related to the higher amount of insoluble particles in the film [44].

The bacterial cellulose film presented a reasonable roughness, which increased with plasma treatment. The treated film showed a more rugged structure.

The gelatin film presented a flat structure with several small dots attributed to insoluble matter. The plasma-treated film presented a different aspect, with a smoother surface. The small dots were drastically reduced, which could be attributed to a better homogenization and interaction of the soluble and insoluble matter.

The films’ hydrophobicity increased more in the films that presented a higher smoothness (starch and gelatin) than in the film (bacterial cellulose) where plasma treatment increased the roughness. The hydrophobicity is linked with the availability and exposure of hydroxyl groups, which are reduced during plasma treatment. Thus, the reduction in hydrophobicity may be associated with the decrease in hydroxyl groups and the changes in surface morphology, which may have reduced the exposure of the remaining hydroxyl groups.

### 3.6. Mechanical Properties

The mechanical properties of the films were evaluated at the best operating conditions for each film (20 min, 50 Hz for starch, 900 Hz for bacterial cellulose and gelatin) and compared to the control (untreated films). Table 8 presents the values obtained for the tensile strength (TS), elongation at break (ER), and elasticity module (EM) for all films.

Plasma treatment increased the mean values of the tensile strength of the films. However, due to the high standard deviation of this kind of test, it is not possible to affirm that plasma treatment has statistically increased the films’ tensile strength. The same trend was observed with the elasticity module, where the mean value increased after plasma treatment. The elongation at break decreased for the starch and gelatin films, while it increased for the bacterial cellulose film. Overall, it is possible to notice that plasma treatment tended to make the films more rigid.

The tensile strength is related to the mechanical resistance of the film. Plasma processing increased the average tensile strength of the films by 41%, 1%, and 23%, respectively, for starch, bacterial cellulose, and gelatin films. Although the mechanical resistances of the films were statistically similar, there was a positive trend in the average value, leading to more resistant starch and gelatin films.

The elongation at break is related to film flexibility. Plasma treatment only increased the flexibility of the bacterial cellulose (by 25%), while a reduction of 29% and 56% was observed in the starch and gelatin films, respectively.

Table 9 presents the mechanical properties of several films reported in the literature. Compared to other techniques, plasma treatment considerably increased the tensile strength of corn starch films. The increase in mechanical resistance attained by plasma treatment was higher than the increase gained with adding natural additives and similar to the increase achieved by high-pressure processing in potato starch films. Thus, mechanical resistance seems to increase more with physical treatments than chemical treatments.

However, the flexibility of starch films decreased with the physical treatment and improved when specific natural products, such as fireweed extract, were incorporated into the film. In most cases, chemical treatments decreased the flexibility of starch films more than physical treatments.

The tensile strength and elongation at break of bacterial cellulose and its composites reported in the literature have a broad range of values (from 1 to 70 MPa for the tensile strength and from 7% to 80% for the elongation at break), which are affected by the formation methodology of the film and other parameters. The methods used herein produced a film with high tensile strength and low elongation at break, while the methods used by Efthymiou et al. [45] and Papadaki et al. [46] produced a film with low tensile strength and high elongation at break. Plasma treatment increased both tensile strength and elongation at break, as occurred with the chemical treatment to convert bacterial cellulose to nanocrystalline cellulose. The amplitude of change of the tensile strength was similar between the plasma and chemical treatments. However, the chemical treatment appears to better affect the elongation at break. The production of composites such as whey protein + bacterial cellulose increases the tensile strength but decreases the elongation at break.

Plasma treatment showed a superior effect in increasing the tensile strength of gelatin films compared to the addition of natural extracts, such as extracts from *B. gymnorhiza* and *S. alba* [53]. However, the effect was lower than that attained by producing a gelatin and polyvinyl alcohol composite [47]. Regarding the elongation at break, plasma treatment reduced this property, making a more rigid film. On the other hand, the addition of natural compounds [53], enzymatic treatment [48], and the composition of polyvinyl alcohol [47] increased the flexibility of gelatin films.

## 4. Conclusions

Plasma treatment improved the hydrophobicity, surface morphology, tensile strength, and elasticity module and reduced the water solubility of starch, bacterial cellulose, and gelatin films. The applied excitation frequency during plasma generation significantly affected the films’ properties and requires optimization for better results. A low excitation frequency (50 Hz) resulted in greater changes in starch films, while a high excitation frequency (900 Hz) induced greater changes in gelatin and bacterial cellulose films.

Films produced on Mylar™ support should have the support removed before subjecting the film to plasma treatment. Treating the films with the support resulted in less properties’ modifications because the changes occurred on a single surface instead of both surfaces.

Plasma treatment is an alternative to improve the hydrophobicity, surface morphology, water resistance, and mechanical properties of biodegradable films. Its performance can be superior, similar, or lower than other options, such as chemical treatment, enzymatic treatment, the addition of chemical compounds, and composites. It has the advantage of not recurring to chemical or biological additives, and no harmful compound remains in the film after plasma application. The treatment’s disadvantage is its limited effect on the mechanical properties of the films since bigger changes could be achieved by producing composites or by chemical addition.

## Figures and Tables

**Table 1:** Width and humidity (mass basis) of the starch, bacterial cellulose, and gelatin films. For each property, values followed by at least one common letter are not different from each other (*p* > 0.05).

Film	Thickness (mm)	Humidity (%)
Starch	0.054 ± 0.002 ^b^	10.2 ± 0.5 ^a^
Bacterial cellulose	0.126 ± 0.011 ^a^	9.6 ± 2.4 ^a^
Gelatin	0.061 ± 0.003 ^b^	12.5 ± 1.2 ^a^

**Table 2:** The angle of contact and water solubility of the starch, bacterial cellulose, and gelatin films treated with dielectric barrier discharge plasma: with and without Mylar™ support. For each property, values followed by at least one common letter are not different from each other (*p* > 0.05).

Plasma Excitation Frequency (Hz)	Contact Angle (°)			Water Solubility (%)		
	Starch	Bacterial Cellulose	Gelatin	Starch	Bacterial Cellulose	Gelatin
With Mylar^TM^ support						
Control	55.7 ± 0.8 ^a^	80.3 ± 0.3 ^a^	69.9 ± 0.4 ^a^	96.9 ± 1.0 ^a^	16.7 ± 1.0 ^b^	34.3 ± 0.4 ^c^
50	55.1 ± 1.0 ^a^	77.9 ± 0.6 ^b^	68.9 ± 0.9 ^a^	96.6 ± 0.1 ^a^	18.5 ± 0.4 ^a^	37.2 ± 0.8 ^a^
400	53.8 ± 0.6 ^b^	78.4 ± 0.2 ^b^	69.7 ± 0.7 ^a^	97.4 ± 0.5 ^a^	17.3 ± 0.2 ^a^	36.3 ± 0.5 ^b^
900	54.2 ± 0.5 ^b^	79.6 ± 1.1 ^a^	70.3 ± 0.5 ^a^	96.2 ± 0.3 ^a^	17.1 ± 0.7 ^b^	35.4 ± 0.8 ^c^
Without Mylar^TM^ support						
Control	55.7 ± 0.8 ^a^	80.3 ± 0.3 ^c^	69.9 ± 0.4 ^c^	96.9 ± 1.0 ^a^	16.7 ± 1.0 ^a^	34.3 ± 0.4 ^b^
50	56.3 ± 1.0 ^a^	79.6 ± 0.3 ^c^	70.6 ± 0.2 ^c^	94.8 ± 0.3 ^b^	15.5 ± 0.3 ^a^	37.9 ± 0.6 ^a^
400	55.1 ± 0.7 ^a^	81.2 ± 0.3 ^b^	72.5 ± 0.8 ^b^	95.4 ± 0.4 ^b^	13.9 ± 0.6 ^b^	34.8 ± 0.5 ^b^
900	55.1 ± 0.4 ^a^	83.0 ± 0.4 ^a^	77.4 ± 0.8 ^a^	97.3 ± 0.2 ^a^	11.3 ± 0.4 ^c^	32.4 ± 0.4 ^c^

**Table 3:** The contact angle of the starch, bacterial cellulose, and gelatin films treated with dielectric barrier discharge plasma at an optimal excitation frequency (50 Hz for the starch film and 900 Hz for the bacterial cellulose and gelatin films) without Mylar™ support. For each property, values followed by at least one common letter are not different from each other (*p* > 0.05).

Processing Time (min)	Starch	Bacterial Cellulose	Gelatin
0	55.7 ± 0.8 ^d^	80.3 ± 0.3 ^c^	69.9 ± 0.4 ^c^
10	57.0 ± 0.6 ^c^	83.0 ± 0.6 ^c^	79.0 ± 0.3 ^b^
15	61.0 ± 0.3 ^b^	84.0 ± 0.3 ^b^	81.0 ± 0.7 ^b^
20	64.0 ± 0.6 ^a^	85.0 ± 0.5 ^a^	84.0 ± 0.3 ^a^

**Table 4:** The contact angles of several starches, bacterial cellulose, and gelatin films reported in the literature.

Film	Contact Angle (°)	Reference
Corn starch	55.7	This work
Corn starch (plasma-treated)	64	This work
Corn starch (high amylose)	55	[36]
Corn starch + cinnamaldehyde	107.4	[36]
Potato starch	24.5	[37]
Potato starch (high-pressure-treated)	30.2	[37]
Banana starch	50.3	[10]
Banana starch (plasma-treated)	65.1	[10]
Gelatin	69.9	This work
Gelatin (plasma-treated)	84	This work
Gelatin	68	[38]
Gelatin + chitosan	56	[38]
Bacterial cellulose	80.3	This work
Bacterial cellulose (plasma-treated)	85	This work
Bacterial cellulose	36	[39]
Bacterial cellulose (plasma-treated)	21	[39]
Bacterial cellulose	14.9	[40]
Bacterial cellulose + curcumin extract	38.6	[40]

**Table 5:** Solubility of the starch, bacterial cellulose, and gelatin films treated with dielectric barrier discharge plasma at an optimal excitation frequency (50 Hz for the starch film and 900 Hz for the bacterial cellulose and gelatin films) without Mylar™ support. For each property, values followed by at least one common letter are not different from each other (*p* > 0.05).

Processing Time (min)	Starch	Bacterial Cellulose	Gelatin
0	96.9 ± 1.0 ^a^	16.7 ± 1.1 ^a^	34.3 ± 0.4 ^a^
10	94.0 ± 0.5 ^b^	11.0 ± 0.8 ^b^	31.0 ± 0.1 ^bc^
15	92.0 ± 0.4 ^c^	10.0 ± 0.3 ^b^	30.0 ± 0.3 ^bc^
20	91.0 ± 0.1 ^c^	10.0 ± 0.2 ^b^	28.0 ± 0.3 ^c^

**Table 6:** Solubility of several starches, bacterial cellulose, and gelatin films reported in the literature.

Film	Water Solubility (%)	Reference
Corn starch	96.6	This work
Corn starch (plasma-treated)	91	This work
Corn starch (high amylose)	17	[36]
Corn starch + cinnamaldehyde	16	[36]
Corn starch	34	[43]
Corn starch + orange peel	30	[43]
Corn starch	19.4	[44]
Corn starch + date palm pits	10.2	[44]
Potato starch	28.1	[37]
Potato starch (high-pressure-treated)	29.6	[37]
Banana starch	37.4	[10]
Banana starch (plasma-treated)	30.7	[10]
Bacterial cellulose	16.7	This work
Bacterial cellulose (plasma-treated)	10	This work
Bacterial cellulose	52.8	[45]
Bacterial cellulose (nanocellulose)	27.3	[45]
Bacterial cellulose (nanocrystalline)	32.5	[45]
Whey protein	43	[46]
Whey protein + bacterial cellulose	38	[46]
Gelatin	34.3	This work
Gelatin (plasma-treated)	28	This work
Gelatin	64	[47]
Gelatin + carboxymethylcellulose	21	[47]
Gelatin + polyvinyl alcohol	8	[47]
Gelatin	35.4	[48]
Gelatin (transglutaminase-treated)	26.6	[48]

**Table 7:** Scores obtained for the desirability function used to select the best starches, bacterial cellulose, and gelatin films’ processing conditions.

Plasma Frequency (Hz)		Scores	
	Starch	Bacterial Cellulose	Gelatin
50	1.36	2.71	2.22
600	1.28	2.73	2.25
900	1.3	2.88	2.71

**Table 8:** Mechanical properties of the starch, bacterial cellulose, and gelatin films treated for 20 min with dielectric barrier discharge plasma at an optimal excitation frequency (50 Hz for the starch film and 900 Hz for the bacterial cellulose and gelatin films) without Mylar™ support. For each property, values followed by at least one common letter are not different from each other (*p* > 0.05).

	Starch	Bacterial Cellulose	Gelatin
Tensile strength (MPa)			
Control	11.25 ± 3.59 ^a^	60.64 ± 5.16 ^a^	28.72 ± 2.41 ^a^
Plasma-treated	15.82 ± 7.79 ^a^	61.32 ± 11.07 ^a^	35.48 ± 6.43 ^a^
Elongation at break (%)			
Control	22.50 ± 3.10 ^a^	7.71 ± 1.39 ^a^	16.10 ± 3.22 ^a^
Plasma-treated	16.04 ± 4.17 ^a^	9.67 ± 2.03 ^a^	7.18 ± 5.94 ^a^
Elasticity module (MPa)			
Control	531 ± 43 ^a^	784 ± 91 ^a^	781 ± 47 ^a^
Plasma-treated	664 ± 61 ^b^	809 ± 62 ^a^	852 ± 101 ^a^

**Table 9:** Mechanical properties of several starches, bacterial cellulose, and gelatin films reported in the literature.

Film	TS	EB	EM	Reference
	(MPa)	(%)	(MPa)	
Corn starch	11.2	22.5	531	This work
Corn starch (plasma-treated)	15.8	16	664	This work
Corn starch (high amylose)	13	43	--	[36]
Corn starch + cinnamaldehyde	14.8	45	--	[36]
Corn starch	0.4	23.5	--	[43]
Corn starch + orange peel	1.4	11.5	--	[43]
Corn starch	3.8	60.1	95	[50]
Corn starch + fireweed extract	4.5	63.6	114	[50]
Corn starch	1.2	53.1	--	[44]
Corn starch + data palm pits	2.9	18.6	--	[44]
Sorghum starch	8.3	1.7	--	[51]
Cassava starch	5	55	--	[50]
Potato starch	4.7	65.2	--	[52]
Potato starch	6.6	19	--	[37]
Potato starch (high-pressure)	11	19	--	[37]
Banana starch	6.2	4.3	144	[10]
Banana starch (plasma-treated)	9.1	7.6	268	[10]
Bacterial cellulose	60.6	7.7	784	This work
Bacterial cellulose (plasma-treated)	61.3	9.6	809	This work
Bacterial cellulose	1.8	33.3	--	[45]
Bacterial cellulose (nanocellulose)	1.7	64.9	--	[45]
Bacterial cellulose (nanocrystalline)	3	77	--	[45]
Bacterial cellulose	125	6.8	--	[40]
Bacterial cellulose + curcumin	45	5	--	[40]
Whey protein	4	30	--	[46]
Whey protein + bacterial cellulose	8	20	--	[46]
Gelatin	28.7	16.1	781	This work
Gelatin (plasma-treated)	35.4	7.2	852	This work
Gelatin	12.2	16.9	--	[53]
Gelatin + *B. gymnorhiza* extract	11.9	17.2	--	[53]
Gelatin	10.4	17.5	--	[53]
Gelatin + *S. alba* extract	13.1	19.4	--	[53]
Gelatin	4	115	--	[47]
Gelatin + carboxymethylcellulose	36	105	--	[47]
Gelatin + polyvinyl alcohol	14	350	--	[47]
Gelatin	65.5	20.4	--	[48]
Gelatin (transglutaminase-treated)	50.6	32.2	--	[48]

## Data Availability

Data are contained within the article.
